# Effect of sclerostin inactivation in a mouse model of severe dominant osteogenesis imperfecta

**DOI:** 10.1038/s41598-023-32221-3

**Published:** 2023-03-27

**Authors:** Juliana Marulanda, Josephine T. Tauer, Iris Boraschi-Diaz, Ghalib Bardai, Frank Rauch

**Affiliations:** 1 Shriners Hospital for Children, 1003 Decarie, Montreal, QC H4A 0A9 Canada; 2 Department of Pediatrics, McGill University, Montreal, QC Canada

**Keywords:** Genotype, Endocrine system and metabolic diseases

## Abstract

Osteogenesis imperfecta (OI) is a rare bone disease that is associated with fractures and low bone mass. Sclerostin inhibition is being evaluated as a potential approach to increase bone mass in OI. We had previously found that in *Col1a1*^Jrt/+^ mice, a model of severe OI, treatment with an anti-sclerostin antibody had a minor effect on the skeletal phenotype. In the present study, we assessed the effect of genetic sclerostin inactivation in the *Col1a1*^Jrt/+^ mouse. We crossed *Col1a1*^Jrt/+^ mice with *Sost* knockout mice to generate *Sost*-deficient *Col1a1*^Jrt/+^ mice and assessed differences between *Col1a1*^Jrt/+^ mice with homozygous *Sost* deficiency and *Col1a1*^Jrt/+^ mice with heterozygous *Sost* deficiency. We found that *Col1a1*^Jrt/+^ mice with homozygous *Sost* deficiency had higher body mass, femur length, trabecular bone volume, cortical thickness and periosteal diameter as well as increased biomechanical parameters of bone strength. Differences between genotypes were larger at the age of 14 weeks than at 8 weeks of age. Transcriptome analysis of RNA extracted from the tibial diaphysis revealed only 5 differentially regulated genes. Thus, genetic inactivation of *Sost* increased bone mass and strength in the *Col1a1*^Jrt/+^ mouse. It appears from these observations that the degree of *Sost* suppression that is required for eliciting a beneficial response can vary with the genetic cause of OI.

## Introduction

Osteogenesis imperfecta (OI) is a heritable condition that is associated with frequent fractures, low bone mass and other skeletal and extraskeletal manifestations^1^. The clinical severity varies widely, from lack of symptoms to perinatal lethality^1,2^. OI is usually caused by dominant mutations in one of the two genes that code for collagen type I, *COL1A1* and *COL1A2*^1,3^. Presently, there is no treatment of the underlying genetic defect, so treatment strategies focus on symptomatic improvements, such as increasing bone mass to reduce bone fragility.

Sclerostin is a secreted molecule that binds to LRP5 on the surface of osteoblasts, whereby it interferes with WNT signaling and thus decreases bone formation^4^. Genetic sclerostin deficiency leads to increased bone formation and sclerotic bone disorders^5^. Pharmacological inhibition of sclerostin using antibodies has been developed to treat disorders with low bone mass, such as postmenopausal osteoporosis^6^, and is also being evaluated for the treatment of OI^7^.

Studies in several mouse models of OI have shown a positive effect of pharmacological sclerostin inhibition on measures of bone mass and bone strength^8–11^. In contrast, we found small effects of sclerostin antibody treatment on the skeletal phenotype of the *Col1a1*^Jrt/+^ mouse, a model of severe OI caused by a dominant splice site mutation in *Col1a1*^12^. One possible explanation for this relative lack of efficacy is that the sclerostin pathway is not important for the control of bone mass in *Col1a1*^Jrt/+^ mice. An alternative possibility is that the dosing of the antibody did not suppress sclerostin sufficiently to increase bone mass in *Col1a1*^Jrt/+^ mice, even though we found in the same study that the dosing regimen increased bone mass and strength in wild type (WT) mice. Finally, the timing of the treatment in the developing vs mature skeleton may account for the limited treatment response in the *Col1a1*^Jrt/+^ mice.

Assessing the effectiveness of sclerostin inhibition in various OI mouse models may have translational implications. Human OI is caused by many different pathogenic variants in *COL1A1*, *COL1A2* and other genes^1^. It is therefore important to elucidate whether targeting the sclerostin pathway is an effective treatment strategy for mice that harbor a range of mutations in OI-associated genes.

The present study aimed to determine whether lifelong genetic sclerostin inactivation is able to improve the bone phenotype of the *Col1a1*^Jrt/+^ mouse model of OI. We therefore crossed the *Col1a1*^Jrt/+^ mice with *Sost* knockout mice^13^ to generate *Sost*-deficient *Col1a1*^Jrt/+^ mice and assessed the resulting skeletal phenotype at different ages.

## Materials and methods

### Mice

All animal work was conducted under approval by the Animal Care Committee at McGill University and conformed to the ethical guidelines of the Canadian Council on Animal Care and ARRIVE guidelines. *Col1a1*^Jrt/+^ mice on FVB background, developed by screening of N-ethyl-N-nitrosourea-induced mutagenesis were a gift from Dr. Jane Aubin’s laboratory, University of Toronto, Canada^14^. These mice have a single nucleotide variant at the splice donor site of *Col1a1* exon 9, leading to skipping of exon 9 and an in-frame loss of 18 amino acids in the N-terminal region of the triple helical domain of the collagen type I alpha 1 chain^14^. The *Sost*^*−/−*^ mice on C57BL6 background were provided by Novartis^13^.

All breeding colonies were maintained at the Animal Care Facility of the Shriners Hospitals for Children-Canada. Mice were housed in groups of up to 5 animals on a 12-h alternating light and dark cycle and had unrestricted access to water and food (Charles River rodent diet 5075, USA).

### Generation of Sost-deficient Col1a1^Jrt/+^ mice

**Figure 1:**
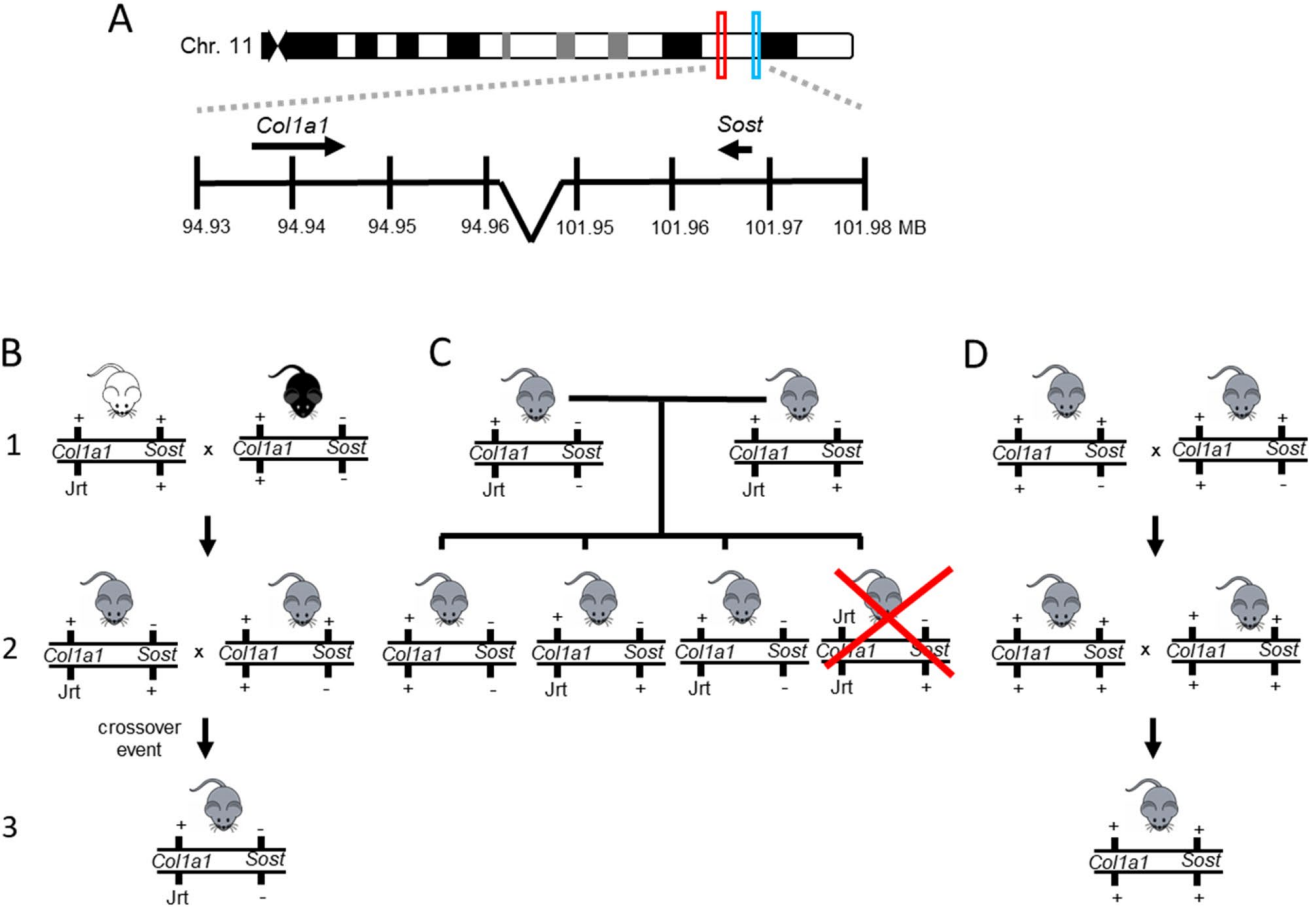
Chromosomal location of *Col1a1* and *Sost* and breeding strategy. (**A**) *Col1a1* and *Sost* are both located on murine chromosome 11, separated by about 7 MB (assembly GRCm38.p6). (**B**) *Col1a1*^*Jrt/*+^ (FVB background) and *Sost*^−/−^ (C57BL6 background) mice were bred (1) to generate *Col1a1*^*Jrt/*+^;*Sost*^+*/−*^ and *Col1a1*^**+***/*+^;*Sost*^+*/−*^ mice of mixed background (2). These mice were then crossed until a crossover event was observed that resulted in the generation of a *Col1a1*^*Jrt/*+^;*Sost*^−/−^ mouse (3). (**C**) *Col1a1*^*Jrt/*+^;*Sost*^−/−^ mice were bred with *Col1a1*^*Jrt/*+^;*Sost*^+*/−*^ mice to generate three of the four genotypes that were used for phenotype analyses. Mice that are homozygous for the Jrt allele (*Col1a1*^*Jrt/Jrt*^) are not viable and die in utero (and are therefore crossed out in the schematic). (**D**) A separate colony was maintained to generate mice of mixed FVB/C57BL6 background that were wild type for both the *Col1a1* and the *Sost* locus. These mice were used to compare results of the three viable genotypic groups shown in (**C**).

The *Col1a1* and *Sost* genes are both located on murine chromosome 11, approximately 7 MB apart (Fig. 1A). To generate *Sost*-deficient *Col1a1*^Jrt/+^ mice, first *Col1a1*^*Jrt/*+^ mice (FVB background) were bred with *Sost*^*−/−*^ mice (C57BL6 background) to generate *Col1a1*^*Jrt/*+^;*Sost*^+*/−*^ and *Col1a1*^+*/*+^;*Sost*^+*/−*^ mice of mixed FVB/C57BL6 background (Fig. 1B)*. Col1a1*^*Jrt/*+^;*Sost*^+*/−*^ and *Col1a1*^+*/*+^;*Sost*^+*/−*^ mice were then crossed until a mouse with a *Col1a1*^*Jrt/*+^;*Sost*^*−/−*^ genotype was generated, indicating that homologous recombination had occurred between the *Col1a1*^*Jrt*^ and the *Sost*^*-*^ locus in that mouse. The colony was subsequently maintained by crossing *Col1a1*^*Jrt/*+^;*Sost*^*−/−*^ mice with *Col1a1*^*Jrt/*+^;*Sost*^+*/−*^ mice from the same generation to keep the contributions of the FVB and C57BL6 backgrounds constant (Fig. 1C). In order to obtain *Col1a1*^+*/*+^;*Sost*^+*/*+^ mice with matching background, a parallel colony with WT mice of mixed FVB/C57BL6 background was established (Fig. 1D). The phenotyping experiments presented here were performed in mice that were obtained after 6 to 8 generations of crossing on mixed background. Results in the following four genotypes were compared: *Col1a1*^+/+^;*Sost*^+/+^ (subsequently called WT), *Col1a1*^+/+^;*Sost*^*−/−*^ (WT;Sost-ko), *Col1a1*^Jrt/+^;*Sost*^+/−^ (Jrt;Sost-het), Col1a1^Jrt/+^;*Sost*^*−/−*^ (Jrt;Sost-ko). The rationale for this breeding strategy was that the main aim of the project was to assess the effect of *Sost* deficiency in *Col1a1*^Jrt/+^ mice. It was therefore important to compare *Sost*-deficient *Col1a1*^Jrt/+^ mice to non-*Sost*-deficient littermates. Previous reports indicated that heterozygous *Sost* deficiency does not have a major effect on the skeleton in mice^15^, which was also observed in the present study (Supplemental Fig. 1).

### Sample collection

For dynamic histomorphometric analyses of the bone, each mouse received two intraperitoneal injections of calcein (20 mg per kg body weight) at 5 days and at 2 days before sacrifice. Mice of both sexes were euthanized either at 8 weeks or at 14 weeks of age. Body weights were recorded at the time of euthanasia. Blood samples were collected by intracardiac puncture, and serum was separated by centrifuge and stored at − 80 °C until analysis. Femoral and tibia lengths were measured with a digital caliper. Right femurs were collected for micro-computed tomography and three-point bending tests. Right femurs were stored at − 20 °C in phosphate buffered saline-soaked gauze until testing or further specimen preparation. Left femurs and the lumbar spines were collected for dynamic histomorphometric analyses. For RNA sequencing, tibias were collected and stored in RNA Later (Invitrogen) at − 80 °C until further processing.

### Micro-computed tomography

Micro-computed tomography of left femurs was performed using a Skyscan 1272 device (Bruker). The voxel size was 5 μm. At the distal femur, trabecular bone was analyzed in a region starting at 0.5 mm proximal of the distal femoral growth plate (to avoid primary spongiosa) and scanning a 1.0 mm section of bone in a proximal direction. Scans were quantified using the system's analysis software (Skyscan CT Analyser, Version 1.16.1.0). To analyze cortical bone at the midshaft femur, scanning was performed starting at 44% of the total femur length from the distal end and scanned for 1 mm proximally. The software derives outer bone diameter (periosteal diameter) and the diameter of the bone marrow cavity (endocortical diameter) from cross-sectional areas using a circular bone cross-section model. Cortical thickness is calculated as the difference of the periosteal and endocortical bone diameter divided by 2.

### Three-point bending test

Following micro-computed tomography scanning, right femurs were loaded to failure in three-point bending using a Materials Testing System Model 5943 (Instron, Norwood, USA). The specimens were thawed 1 day prior to the test and all muscle tissues were cleaned off. The bone was soaked overnight in phosphate buffered saline at room temperature until mechanical testing. The distance between the lower supports was 7 mm with a vertical displacement rate of 50 μm/s. The anterior mid-diaphysis was loaded under tension and the tests were analyzed using the system’s analysis software Bluehill (Illinois Tool Works Inc., Glenview, USA; Version 3.65).

### Serum bone markers

Serum levels of the bone formation marker procollagen type I N-terminal propeptide (PINP; Mouse/Rat PINP, Immunodiagnostic Systems) and the bone resorption marker tartrate-resistant acid phosphatase 5b (TRAcP5b, Mouse TRAP, Immunodiagnostic Systems) were quantified by enzyme immunoassays. Serum tests were performed in duplicate.

### Bone histomorphometry

Histomorphometric analysis of trabecular bone were performed at the left distal femur (starting at 50 μm proximal to the growth plate to a distance of 1.4 mm from the growth plate) and in lumbar vertebra 4 (L4, entire trabecular compartment). Specimens were fixed in 10% phosphate-buffered formalin, dehydrated in increasing concentrations of ethanol and embedded in methylmethacrylate. Undecalcified 6 μm thick sections were cut with a Polycut E microtome (Reichert-Jung, Heidelberg, Germany). The sections were deplastified with ethylene glycol monoethyl acetate to allow for optimal staining. In each sample, two consecutive sections were selected that were stained with Masson Goldner Trichrome for static parameters or mounted unstained for the measurement of dynamic parameters using fluorescence microscopy. Histomorphometric measurements were carried out using a digitizing table with Osteomeasure^®^ software (Osteometrics Inc., Atlanta, GA, USA). Nomenclature and abbreviations follow the recommendations of the American Society for Bone and Mineral Research^16^.

### RNA sequencing

Male mice were euthanized at the age of 8 weeks (*n* = 4 mice per group). Tibia shafts were dissected, the bone marrow was flushed out and the bones were immediately immersed in RNAlater (Thermo Fisher) and stored at − 80 °C until processing. After mincing with scissors, the bones were blotted to remove excess RNAlater and manually grinded using a liquid nitrogen-cooled mini mortar and pestle set, and then transfered to 5 ml of TRIzol. The total RNA was extracted using the phenol–chloroform method.

Library preparation, RNA sequencing, data post-processing and statistical evaluation were performed as described^17^. The total RNA samples used in the present study had a concentration between 78 and 389 ng/μL, with an RNA integrity number ranging between 7.4 and 8.4. Libraries were generated from 250 ng of total RNA. Sequencing was performed on a NovaSeq S4 (Illumina) for 2 × 100 cycles (paired-end mode). Between 31 and 57 million 100-base paired-end reads were generated per sample.

Analysis of paired-end sequencing reads was performed using GenPipes, an open-source, Python-based standardized analysis pipeline, hosted on the Compute Canada High Performance Computing center (https://www.computecanada.ca). Read pairs were aligned to GRCm38 by the RNA-seq aligner STAR^18^. Aligned RNA-seq reads were assembled into transcripts, and their relative abundance was estimated using Cufflinks^19^ and Cuffdiff^20^. Differential expression analysis was conducted using DESeq^21^. Significantly differentially expressed genes were defined as those with an adjusted *p* value < 0.05 as determined by DESeq.

### Statistical analyses

All data shown in this report are mean ± standard error. Differences between genotypes were assessed for significance by one‐way ANOVA of T-Test when applicable. Post-hoc tests of differences between individual groups were performed with Sidak’s tests for multiple comparisons using GraphPad Prism version 5.02 (GraphPad Software for Windows, San Diego, CA, USA). A p value < 0.05 was considered significant.

## Results

**Figure 2:**
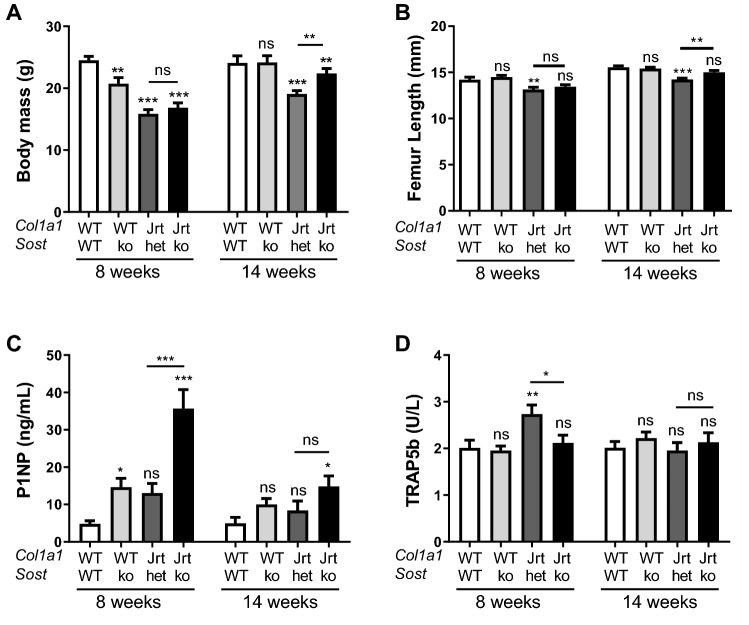
Body mass, femur length and serum markers of bone metabolism. Variation with genotype and age in body mass, femur length, and serum markers of bone metabolism. Error bars represent standard errors. Significance levels for differences to WT mice (ANOVA) are indicated above each bar: *p < 0.05, **p < 0.01, ***p < 0.001, ns: not significant (p ≥ 0.05). Significance levels for differences between Jrt;Sost-het and Jrt;Sost-ko are indicated above the horizontal lines. n = 8–15 mice per group for body mass and femur length. n = 6–11 mice per group for serum markers.

Phenotypes of WT, WT;Sost-ko, Jrt;Sost-het and Jrt;Sost-ko mice were assessed at 8 and at 14 weeks of age. We did not find a significant difference in bone parameters between male and female Jrt;Sost-het and Jrt;Sost-ko mice and therefore results from both sexes were analyzed as one group (Supplemental Fig. 2). Body weight and femur length were lower in Jrt;Sost-het mice than in WT mice at both time points (Fig. 2A,B). Jrt;Sost-ko mice had similar body mass and femur length as Jrt;Sost-het mice at 8 weeks of age, but by the age of 14 weeks results were higher in Jrt;Sost-ko mice. Jrt;Sost-ko mice had higher P1NP serum concentrations and lower TRAP5b serum levels than Jrt;Sost-het mice at the age of 8 weeks, whereas no significant biomarker differences between these two genotypes were found at 14 weeks of age (Fig. 2C,D). Serum levels of alkaline phosphatase, calcium and phosphorus were similar between Jrt;Sost-het and Jrt;Sost-ko mice, at both 8 and 14 weeks of age (Supplemental Fig. 3).

**Figure 3:**
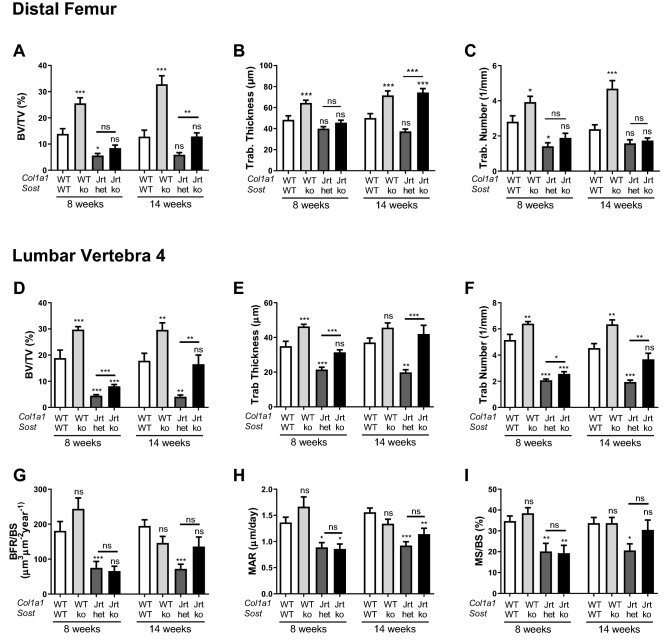
Trabecular bone characterization. Trabecular bone analyses at the distal femur (**A**–**C** by microCT) and at lumbar vertebra 4 (**D**–**I** by histomorphometry). Error bars represent standard errors. Significance levels for differences to WT mice (ANOVA) are indicated above each bar: * p < 0.05, **p < 0.01, ***p < 0.001, ns: not significant (p ≥ 0.05). Significance levels for differences between Jrt;Sost-het and Jrt;Sost-ko are indicated above the horizontal lines. n = 6–12 mice per group for femur analyses and 11–15 mice per group for vertebra analyses.

**Figure 4:**
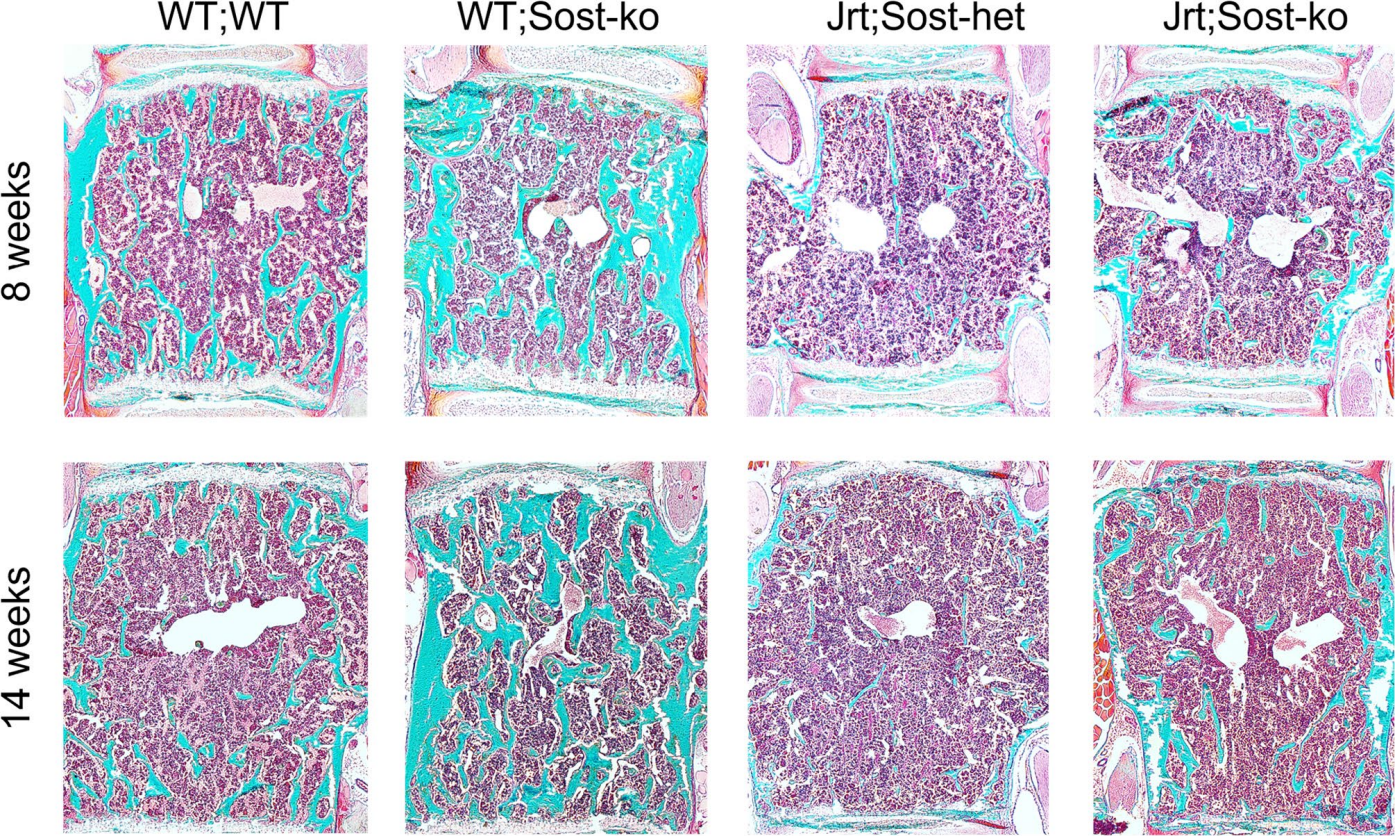
Histology of vertebral bones. Histological longitudinal sections of lumbar vertebra stained with Goldner’s trichrome staining at 8 and 14 weeks of age. Mineralized bone appears blue, unmineralized bone pink and bone marrow purple.

Regarding trabecular bone, WT;Sost-ko mice had higher bone volume per tissue volume than WT mice, as expected (Fig. 3). Jrt;Sost-ko mice had higher trabecular bone volume than Jrt;Sost-het mice at both skeletal sites, but the difference between these two genotypes was larger at 14 weeks than at 8 weeks of age. At the distal femur, the higher trabecular bone volume in Jrt;Sost-ko mice was entirely due to higher trabecular thickness (Fig. 3A–C), whereas in vertebra both trabecular thickness and number were higher (Figs. 3D–F and 4). Dynamic bone histomorphometry at the lumbar vertebra 4 showed that bone formation rate was lower in Jrt;Sost-het mice than in WT mice, but no difference in bone formation rate was observed between Jrt;Sost-het and Jrt;Sost-ko mice (Fig. 3G–I).

**Figure 5:**
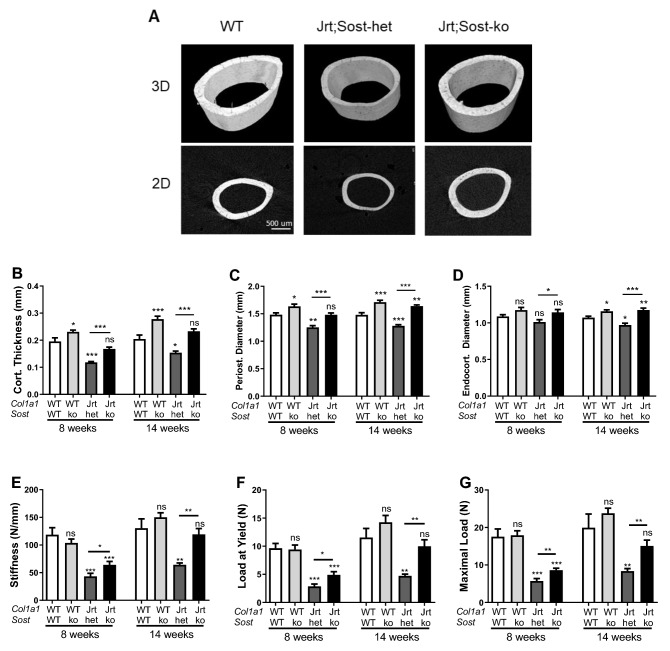
Cortical bone characterization. Femur midshaft microCT and biomechanics. (**A**) MicroCT images of midshaft femur scans. (**B**–**D**) MicroCT data for bone structure. (**E**–**G**) Results of three-point bending tests. Error bars represent standard errors. Significance levels for differences to WT mice (ANOVA) are indicated above each bar: *p < 0.05, **p < 0.01, ***p < 0.001, ns: not significant (p ≥ 0.05). Significance levels for differences between Jrt;Sost-het and Jrt;Sost-ko are indicated above the horizontal lines. n = 6–15 mice per group.

As to cortical bone at the midshaft femur, Jrt;Sost-het mice had thinner cortices, smaller periosteal diameter, and, at 14 weeks, smaller endocortical diameter than WT mice. Jrt;Sost-ko mice had thicker cortices, and larger periosteal and endocortical diameters than Jrt;Sost-het mice (Fig. 5A–D). Correspondingly, Jrt;Sost-ko mice had higher biomechanical parameters of bone strength than Jrt;Sost-het mice (Fig. 5E–G). These differences in femur diaphyseal anatomy and strength between Jrt;Sost-ko mice and Jrt;Sost-het mice tended to be larger at 14 weeks than at 8 weeks of age.

We next extracted RNA from the tibia diaphysis of Jrt;Sost-het and Jrt;Sost-ko to assess the effect of sclerostin deficiency on the transcriptome. Differential gene expression analysis confirmed that Jrt;Sost-ko mice were deficient in *Sost* on the mRNA level (Table 1). Apart from *Sost*, only three other genes were downregulated (*Colgalt2*, *Tex15*, *Pla1a*) and one gene was upregulated (*Pla2g5*) in Jrt;Sost-ko mice compared to Jrt;Sost-het mice.

**Table 1:** List of differentially regulated genes in Jrt;Sost-ko mice compared to Jrt;Sost-het mice.

Gene	Description	Log2 fold change	P
Downregulated			
* Sost*	Sclerostin	− 5.88	< 0.001
* Colgalt2*	Collagen betagalactosyltransferase 2	− 1.27	0.01
* Tex15*	Testis expressed gene 15	− 0.51	0.01
* Pla1a*	Phospholipase A1 member A	− 1.16	0.03
Upregulated			
* Pla2g5*	Phospholipase A2, group V	0.59	0.03

P values are adjusted by the Benjamini–Hochberg procedure, as determined by Deseq.

N = 4 mice per group.

## Discussion

In this study we found that genetic *Sost* inactivation had marked effects on the skeletal phenotype in the *Col1a1*^Jrt/+^ mouse model of OI. Compared to Jrt;Sost-het mice, Jrt;Sost-ko mice had higher body mass, femur length, trabecular bone volume, cortical thickness and periosteal diameter as well as increased biomechanical parameters of bone strength. The effect of sclerostin deficiency in *Col1a1*^Jrt/+^ mice seemed to increase with age, as differences between Jrt;Sost-ko and Jrt;Sost-het mice were larger at 14 weeks than at 8 weeks of age.

In our previous studies, we had found only small effects of sclerostin antibody treatment in the *Col1a1*^Jrt/+^ mouse^12^. It was therefore not clear whether the sclerostin pathway was important for the control of bone mass in this mouse model of severe OI. The present study demonstrates that complete sclerostin inactivation does have a beneficial effect in the *Col1a1*^Jrt/+^ mouse.

The effect size of *Sost* inactivation seemed broadly similar between *Col1a1*^Jrt/+^ and WT mice. At the femur of 14-week-old mice, *Sost* inactivation was associated with more than twice higher trabecular bone volume in both *Col1a1*^Jrt/+^ and WT mice, and an approximately 50% higher cortical thickness. The changes associated with genetic *Sost* depletion compare favorably to the previously reported effects of 4 weeks of sclerostin antibody treatment in *Col1a1*^Jrt/+^ mice, where a 65% increase in trabecular bone volume and an 18% increase in cortical thickness was found in growing mice^12^.

It therefore appears that targeting sclerostin is an effective approach also in *Col1a1*^Jrt/+^ mice but that presumably our previous antibody treatment did not suppress sclerostin sufficiently to elicit stronger positive effects. It should be noted, however, that the same dosing regimen had been able to induce a robust bone response in littermate WT mice. Other investigators have reported that sclerostin antibody treatment has a marked beneficial effect in a range of OI mouse models^8–11^. Overall, these results suggest that the response to pharmacological sclerostin inhibition may vary with the specific disease-causing mutation. It is therefore possible that in some circumstances a more profound suppression of sclerostin is required to achieve the desired effects.

We observed that *Sost* inactivation in *Col1a1*^Jrt/+^ mice had larger effects at 14 weeks of age than at 8 weeks of age. This is in accordance with the initial description of the Sost-ko mouse used in the present project, which showed that bone mass was similar to WT mice up to the age of 1 month, but subsequently kept increasing continuously, even after mice reached sexual maturity^13^. Later studies found that, compared to WT mice, Sost-ko mice kept gaining both trabecular and cortical bone mass from 10 to 26 weeks of age^22,23^. The reasons why constitutive inactivation of *Sost* leads to increased bone mass only relatively late in skeletal development are not clear. However, recent data suggest that bones in 12-week old Sost-ko mice continue to undergo modeling, whereas in Sost-het mice of the same age only remodeling occurs^24^. As modeling consists of bone formation without prior resorption, modeling quickly increases bone mass^25^. This might explain why *Sost* inactivation leads to continuing accumulation in bone mass even at an age when bone mass has reached a plateau in *Sost* replete mice.

It is interesting to note that in 8-week old *Col1a1*^Jrt/+^ mice, *Sost* deficiency was associated with a marked increase in circulating levels of the bone formation marker P1NP whereas trabecular bone formation rate as measured by histomorphometry was unchanged. We had made similar observations in *Col1a1*^Jrt/+^ mice treated with sclerostin antibodies^12^. The causes of this discrepancy were not investigated, but P1NP is a systemic marker of bone formation and therefore is influenced by the speed of bone growth in length, cortical bone growth in width and trabecular bone remodeling in the entire skeleton, whereas histomorphometry as measured in the present study reflects only trabecular remodeling in lumbar vertebra 4. In addition, P1NP is released from collagen type I molecules by N-propeptide cleavage after procollagen secretion, whereas histomorphometric bone formation rate measures the amount of bone deposited in trabecular tissue. As *Col1a1*^Jrt/+^ mice have a mutation in the *Col1a1* gene, it is possible that a proportion of the procollagen molecules that are generated in these mice are not integrated into the bone. This view is also in accordance with the observation that *Sost* deficiency did not alter serum levels of alkaline phosphatase, a marker of bone formation that is not directly influenced by *Col1a1* mutations.

Our RNA sequencing results showed surprisingly few differentially regulated genes between Jrt;Sost-ko mice and Jrt;Sost-het mice, despite the significant differences in their skeletal phenotypes. The transcriptome analysis did confirm that *Sost* expression was absent in Jrt;Sost-ko mice, but among the other differentially regulated genes, only *Colgalt2* has an obvious link to collagen metabolism or OI. *Colgalt2* codes for an enzyme that glycosylates hydroxylated lysine residues in collagen, which may be important for collagen secretion^26^. However, it is unclear whether downregulation of *Colgalt2* contributes to the phenotypic differences between Jrt;Sost-ko mice and Jrt;Sost-het mice that we found in the present study.

A limitation of the present study is that we were unable to compare Jrt;Sost-ko mice to Jrt;Sost-WT littermates. This was due to the fact that the *Col1a1* and *Sost* genes are located in proximity on mouse chromosome 11 and are therefore in close linkage disequilibrium. We therefore compared Jrt;Sost-ko to Jrt;Sost-het mice. Previous studies have found that heterozygous *Sost* inactivation does not cause a bone phenotype in mice^15^, as also observed here. However, in humans heterozygous inactivating *Sost* mutations are associated with slightly higher bone mass^27^. If heterozygous *Sost* deficiency in fact had a similar effect in *Col1a1*^Jrt/+^ mice as it has in humans, then our data would underestimate the effect of homozygous *Sost* deficiency in *Col1a1*^Jrt/+^ mice. An additional limitation is the low sample size used in the RNA seq experiment. As in any statistical evaluation, a larger sample size is likely to identify a larger number of statistically significant differences between groups.

In conclusion, the present study shows that complete inactivation of *Sost* increases bone mass and strength in the *Col1a1*^Jrt/+^ mouse, even though pharmacological *Sost* inhibition had previously shown only minimal effects in this mouse model of OI. These observations suggest that the degree of Sost suppression that is required for eliciting a beneficial response can vary with the genetic cause of OI.

## Supplementary Information

Supplementary Figures.

## Data Availability

The datasets generated and analyzed during the current study are available in the Gene Expression Omnibus (GEO) repository, accession number GSE220601 and link: https://www.ncbi.nlm.nih.gov/geo/query/acc.cgi?acc=GSE220601.
