# Supplementary material for: Effect of sclerostin inactivation in a mouse model of severe dominant osteogenesis imperfecta
Source: Sci Rep. 2023 Mar 27;13:5010. doi: 10.1038/s41598-023-32221-3 (PMC10043013; doi:10.1038/s41598-023-32221-3)
Supplement: Supplementary file 1 — Supplementary Figures. [file 41598_2023_32221_MOESM1_ESM.docx]

**Supplemental Material**

**Supplemental Figure 1:** MicroCT analysis of trabecular bone at the distal femur (A-C) and of cortical bone at the femur midshaft (D-F) in 8-week-old WT Sost-replete mice and in WT with heterozygous Sost knockout. Error bars represent standard errors. Significance levels for differences between WT;WT and WT;Sost-het are indicated above the horizontal lines. ns: not significant (p≥0.05). N= 11 mice per group.

**Supplemental Figure 2:** MicroCT analysis of trabecular bone at the distal femur (A-C) and femur midshaft (D-F) in male and female mice. Error bars represent standard errors. Significance levels for differences between males and females are indicated above the horizontal lines (t-tests): * p<0.05, ***p<0.001, ns: not significant (p≥0.05). n= 4-6 mice per group.

**Supplemental Figure 3:** Serum levels of alkaline phosphatase, calcium and phosphorus in the Jrt;Sost-het and Jrt;Sost-ko mice at 8 and 14 weeks. Error bars represent standard errors. Significance levels for differences between the two genotypes are indicated above the horizontal lines. ns: not significant (p≥0.05). N= 11-15 mice per group.
